# Analysis of the Effect of Pupil Size and Corneal Aberration on the Optical Performance of Premium Intraocular Lenses

**DOI:** 10.3390/jcm14155336

**Published:** 2025-07-29

**Authors:** Juan J. Miret, Vicente J. Camps, Celia García, Maria T. Caballero, Antonio Sempere-Molina, Juan M. Gonzalez-Leal

**Affiliations:** 1 Department of Optics, Pharmacology and Anatomy, University of Alicante, 03690 San Vicente del Raspeig, Alicante, Spain; jjmiret@ua.es (J.J.M.); c.garcia@ua.es (C.G.); mt.caballero@ua.es (M.T.C.); antoniosemperemolina@gmail.com (A.S.-M.); 2 Department of Condensed Matter Physics, Faculty of Sciences, University of Cadiz, 11510 Puerto Real, Cádiz, Spain; juanmaria.gonzalez@uca.es

**Keywords:** cataract, refractive surgery, premium IOLs, Lasik surgery

## Abstract

**Background/Objectives**: To assess the optical performance of two refractive premium IOLs across pupil sizes and values of corneal spherical aberration (SA). **Methods**: Two refractive IOLs were evaluated in this study: Tecnis Eyhance and Mini Well. The surface profiles were obtained to calculate the through-object MTF (TO MTF) curves and simulate optotype images. Entrance pupil sizes ranging from 2 to 5.5 and three corneal models were analyzed in the simulation: an average population aberrated cornea, an aberration-free cornea and a post-Lasik myopic cornea. **Results**: For Model 1 and pupil sizes between 3.0 and 3.5 mm, Mini Well provided acceptable visual quality from far to near distances, whereas Eyhance struggled to maintain visual quality at distances closer than intermediate. For patients with lower-than-normal corneal SA (i.e., more prolate corneas, such as post-hyperopic LASIK) both IOLs exhibited a hyperopic shift in far focus. Conversely, for patients with higher-than-normal corneal SA (i.e., more oblate corneas, such as post-myopic LASIK), the shift occurred in the myopic direction. Despite the implementation of an optimized IOL power to circumvent any shift, the TO MTF nevertheless reflected the interaction between corneal and IOL SA. Furthermore, the Mini Well demonstrated increased tolerance to less negative SA values, while Eyhance exhibited behavior consistent with a monofocal lens for more positive SA values. **Conclusions**: Surgeons should consider each patient’s corneal asphericity and typical pupil diameter when selecting and calculating the power of the premium IOLs studied, particularly in patients with a history of refractive surgery.

## 1. Introduction

Recent advancements in intraocular lens (IOL) technology have led to the development of premium IOLs that provide either multiple focal points or an extended depth of focus (EDOF). These lenses utilize refractive or diffractive optics and incorporate wavefront-based surface designs to enhance visual performance. It is well established that pupil size and corneal spherical aberration (SA) significantly influence the optical performance of IOLs, particularly those with high-order aspherical surfaces designed for multifocality or EDOF.

According to ISO standards [1], in vitro evaluation of multifocal IOLs should be carried out under two types of corneal conditions—an aberration-free cornea and an average-aberrated cornea—and at two pupil sizes, specifically 3 mm and 4.5 mm at the IOL plane. Multiple investigations, both clinical and in vitro, have highlighted how pupil size [2,3] and corneal SA [4,5,6] significantly affect postoperative outcomes for patients receiving these lenses. Studies have shown that performance of multifocal IOLs may be reduced if the patient’s pupil is notably smaller or larger than typical during routine activities [7]. Likewise, corneal SA has received considerable attention, especially in individuals with a history of refractive surgery [8,9]. Myopic and hyperopic laser surgery alter corneal asphericity, modifying SA and impacting IOL performance [10]. Procedures like myopic or hyperopic LASIK can considerably alter the shape of the cornea, modifying its asphericity and SA. Because many premium IOLs rely on particular SA profiles for optimal function, changes in corneal SA can lead to unexpected or suboptimal visual outcomes if not carefully factored into lens selection and power calculation.

Current evidence remains insufficient for a comprehensive evaluation of multifocal IOL performance, and unexpected outcomes may arise. In prior studies, we introduced a profilometry-based methodology [11] to determine the through-object modulation transfer function (TO MTF) curves for five premium multifocal IOLs. Our findings indicated that optical performance varied with pupil size and corneal SA, with pupil size affecting the TO MTF curve shape and SA changes shifting the far focus [12].

These findings underscored the need for further research across a broader range of pupil sizes and corneal aberration models. The objective of this study was to assess the optical performance of two refractive premium IOLs across pupil sizes ranging from 2 mm to 5.5 mm in three different eye models. These conditions simulate real-world clinical scenarios, including patients with small (2 mm) or large (5.5 mm) pupils. Additionally, an eye model incorporating a corneal SA deviation from ISO standards was introduced to better reflect clinical conditions. A main statement regarding the interaction between the SA of the cornea and the intraocular lens (IOL) for optimized IOL power is further developed in the Discussion section. The details of this methodology are provided in the following sections.

## 2. Materials and Methods

### 2.1. Intraocular Lenses

Two refractive IOLs of 20 D were evaluated in this study: the enhanced monofocal Tecnis Eyhance IOL and the EDOF Mini Well IOL.

The Mini Well (SIFI, Lavinaio, Italy) is an EDOF lens with an optical design featuring three annular zones of alternating SA signs. The Tecnis Eyhance (Johnson & Johnson Vision, Inc., Santa Ana, CA, USA) is a one-piece posterior chamber lens with a spherical posterior surface and a modified aspheric anterior surface. These IOL designs extend depth of focus (DOF) to enhance intermediate vision.

### 2.2. Surface Measurement Method

The three-dimensional (3D) surface profile of each IOL was obtained using confocal grid structured illumination with a multimode optical profilometer (Zeta Instruments, model Z 300, San Jose, CA, USA). Raw data were smoothed using custom routines and algorithms developed in MATLAB version 202 R2 (The MathWorks, Natick, MA, USA). An application programming interface (API) linked MATLAB with Zemax (Zemax OpticStudio, Zemax LLC., Kirkland, WA, USA), enabling ray tracing and subsequent optical analysis [11].

### 2.3. Pupil Sizes and Eye Models

To evaluate IOL optical performance under different luminance conditions, entrance pupil sizes ranging from 2 to 5.5 in 0.5 mm increments were analyzed. In the models, a 3.5 mm entrance pupil corresponded to a 3 mm pupil at the IOL plane, and 5.5 mm corresponded to 4.6 mm. This range enabled evaluation of IOL performance under various lighting conditions reflecting individual pupil size variations.

Using ISO standards and the Liou-Brennan eye model parameters [13], three corneal models were simulated:

- Model 1: Mean population aberrated cornea. This model represents a typical corneal profile. The first corneal surface had an asphericity (Q) of −0.18 for 6 mm of entrance pupil. This configuration resulted in a primary SA ($Z_{0}^{4}$) of +0.26 μm for a 6 mm entrance pupil.
- Model 2: Aberration-free cornea. Designed to isolate IOL performance, this model eliminated corneal SA. The first corneal surface was modified to a more prolate asphericity (Q = −0.57), producing zero SA ($Z_{0}^{4}$ = 0 μm) for a 6 mm pupil entrance. This configuration also represents patients with naturally more prolate corneas or those who have undergone hyperopic LASIK. For reference, a +4.00 D hyperopic correction typically induces an asphericity change (ΔQ) of approximately −0.40, depending on the laser system and ablation profile [14,15].
- Model 3: Myopic LASIK cornea. To complete the analysis, a third model was generated to represent patients with a more oblate cornea than normal, similar to the changes induced by myopic LASIK surgery. An asphericity change (ΔQ) of +0.48 was assumed (Q = +0.3), corresponding to a correction between −4 D and −5 D [16]. This configuration resulted in a $Z_{0}^{4}$ value of +0.62 μm for a 6 mm entrance pupil.

### 2.4. Through-Object MTF Curve Calculation Process

The TO MTF curve was obtained by calculating the MTF value at 50 cycles/mm while varying the object vergence from −4 D to +2 D. As described in a previous study [12], the TO MTF reference plane was the corneal vertex, while for through-focus MTF, it was the IOL plane. Both curves were approximately linked using power translation equations. Standards for clinical studies involving IOLs describe monocular visual acuity defocus curves as one of the most relevant psychophysical metrics. The TO MTF correlates more closely with this clinical parameter because it is measured in the object vergence [12].

**Figure 1:**
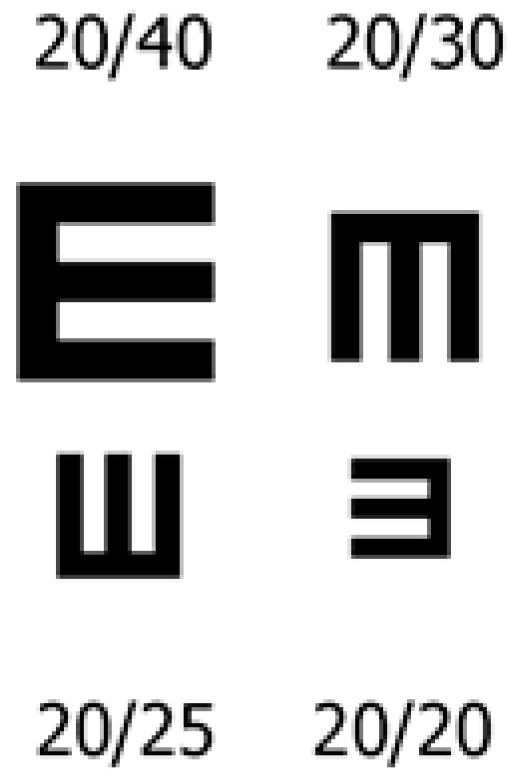
Chart of optotypes and the corresponding simulated visual acuities. The printed “E” is not to scale.

To support findings from the TO MTF curves, simulated optotype images were generated. They included four optotypes for visual acuities (VA) from 20/20 (LogMAR = 0) to 20/40 (LogMAR = 0.3). Optotypes were computed by convolving the optical system (eye + IOL) point spread function (PSF) on the retina with the chart image (Figure 1).

The retinal position was estimated as the one where the MTF value at 50 cycles/mm, for a distant object, reached its maximum. This analysis was performed for Model 1 under a 3.5 mm entrance pupil. To evaluate the impact of pupil size, the TO MTF curve was calculated, and optotypes were simulated for near vision (40 cm), two intermediate distances (60 cm and 90 cm), and far vision. In Models 2 and 3, the axial length was kept constant, matching that of Model 1, to investigate eyes with the same biometric characteristics but with non-standard corneal asphericity values. Only far vision optotypes were analyzed for these two models.

## 3. Results

### 3.1. Mini Well

**Figure 2:**
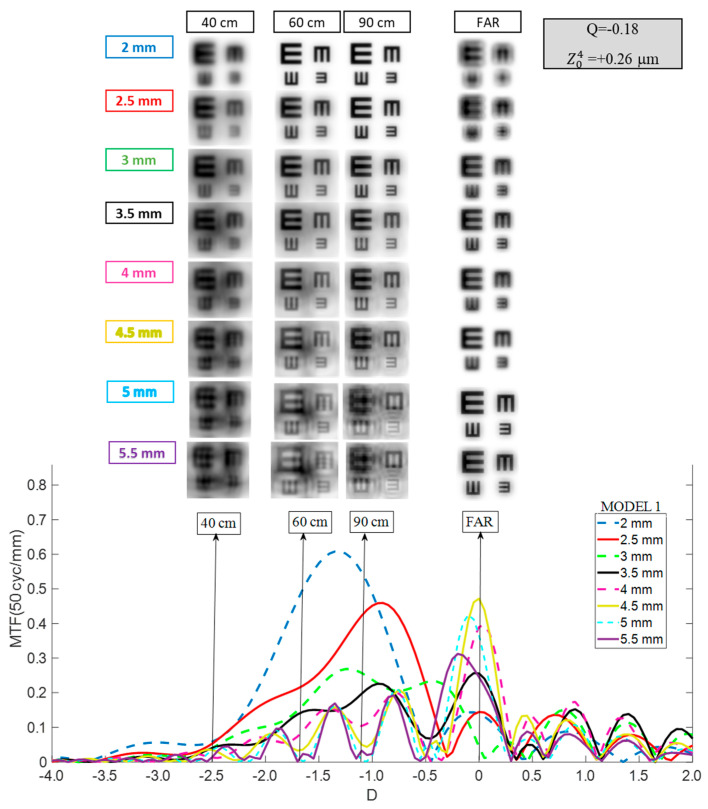
Pupil size dependence of the Mini Well TO MTF curve considering Model 1. Simulated optotypes are shown for all pupil sizes and distances.

In Model 1 (Figure 2), the TO MTF shape varied with pupil size. For entrance pupils smaller than 3 mm, it exhibited a single broad peak, forming a plateau extending into intermediate and near vision. However, the pronounced myopic shift of approximately −1 D led to a reduction in far vision performance.

Between 3 mm and 4.5 mm, the MTF curves split into two regions. The first, similar to smaller pupils, featured a broad plateau that gradually declined into negative defocus (intermediate and near vision). The second, centered around far vision (0 D), displayed the highest peak. The far vision peak reached its maximum for a pupil of 4.5 mm, decreasing for larger pupils. Meanwhile, the plateau for intermediate and near vision became increasingly fragmented into multiple peaks as pupil size increased.

For far vision, 20/20 optotypes were discernible for pupil sizes between 3 mm and 5.5 mm. For smaller pupils, contrast was reduced due to the diminished MTF at the far peak. At intermediate distances (60 cm and 90 cm), 20/20 optotypes were recognizable across all pupil sizes, although contrast declined with larger pupils, particularly at 5 mm and 5.5 mm. At a near distance (40 cm), 20/20 optotypes were identifiable for sizes between 2.5 mm and 3.5 mm, while 20/25 optotypes remained recognizable up to 4 mm.

**Figure 3:**
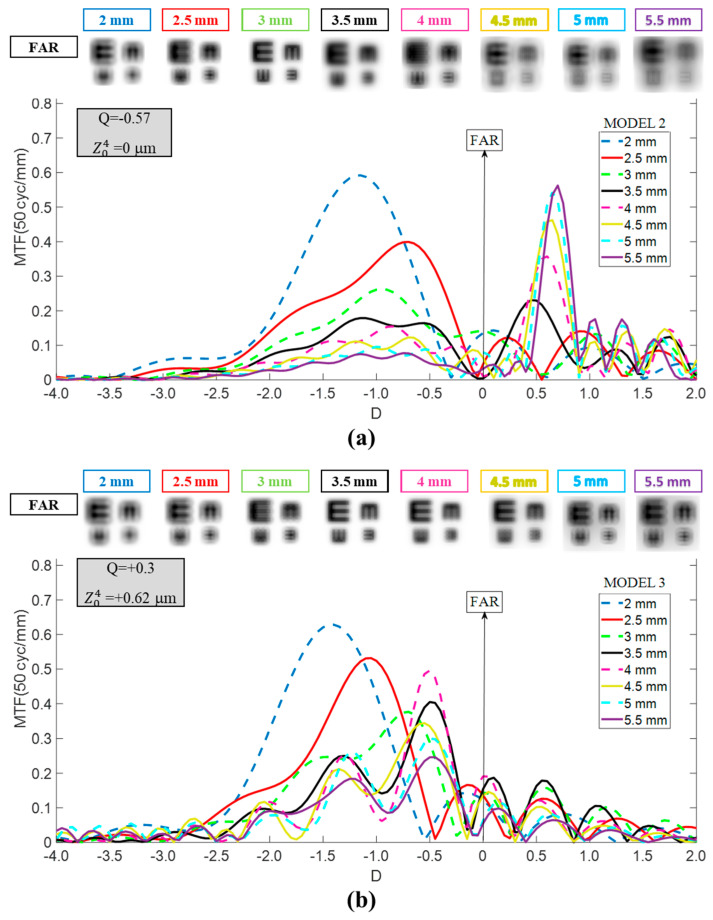
Dependency of the Mini Well TO MTF curves on the pupil size for Models 2 (**a**) and 3 (**b**). Simulated optotypes for far vision and all pupil sizes are shown.

The TO MTF curves for Models 2 and 3 are shown in Figure 3. For Model 2 (cornea with zero SA at 6 mm entrance pupil), the curve shifted toward the hyperopic zone relative to Model 1, particularly for pupil diameters exceeding 3 mm, by +0.5 D to +0.7 D. In contrast, Model 3 (cornea with SA of +0.62 μm at 6 mm entrance pupil) exhibited a myopic shift of approximately −0.5 D for pupil sizes over 3 mm, along with a marked narrowing of the TO MTF curve, which reduced the dioptric range between its peak and the point where it approaches zero. Both myopic and hyperopic displacements caused a deterioration of distance vision, especially for Model 2.

### 3.2. Tecnis Eyhance

**Figure 4:**
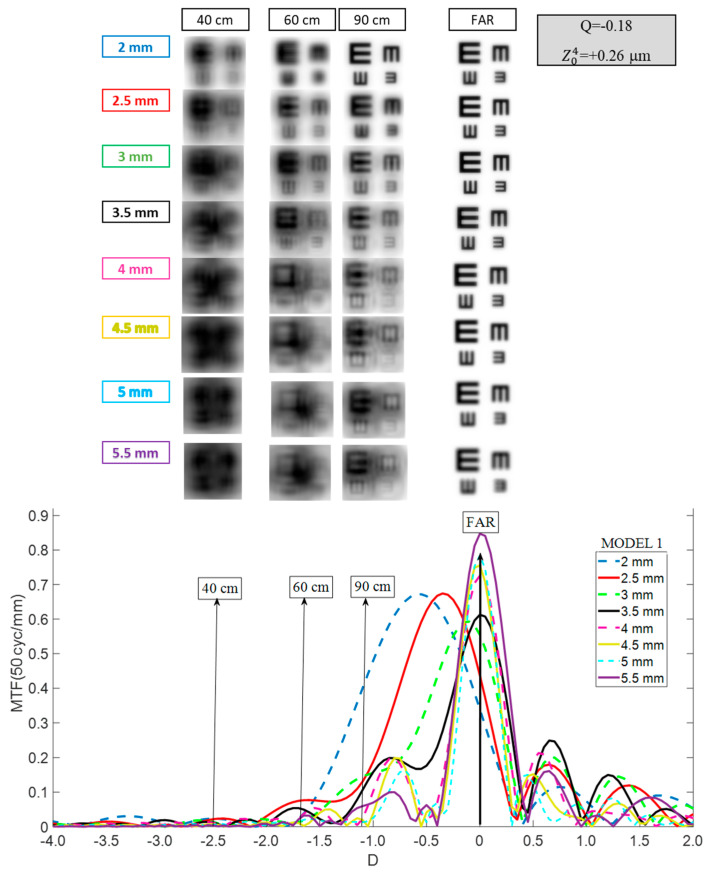
Pupil size dependence of the Tecnis Eyhance TO MTF curve considering Model 1. Simulated optotypes are shown for all pupil sizes and distances.

As shown in Figure 4 with a 2 mm entrance pupil, the curve exhibited a single broad peak centered at −0.6 D (myopic shift), extending from far to intermediate vision. Between 2.5 mm and 3.5 mm, this shift declines, with the peak centering at 0 D (far vision) for a 3.5 mm pupil. Simultaneously, a hump or plateau emerged on the negative side of the peak reaching almost to the end of the intermediate zone (60 cm). For larger pupils, the far vision peak remained stable and even contributed to enhanced optical quality. However, as size increased, the area under the maximum peak became narrower, resembling a monofocal IOL.

A 20/20 VA was consistently achieved at far vision, regardless of pupil size. However, at 90 cm, acceptable performance was limited to pupils up to 3.5 mm, and at 60 cm, recognition was only possible for the 2.5 mm and 3 mm pupils, albeit with difficulty.

**Figure 5:**
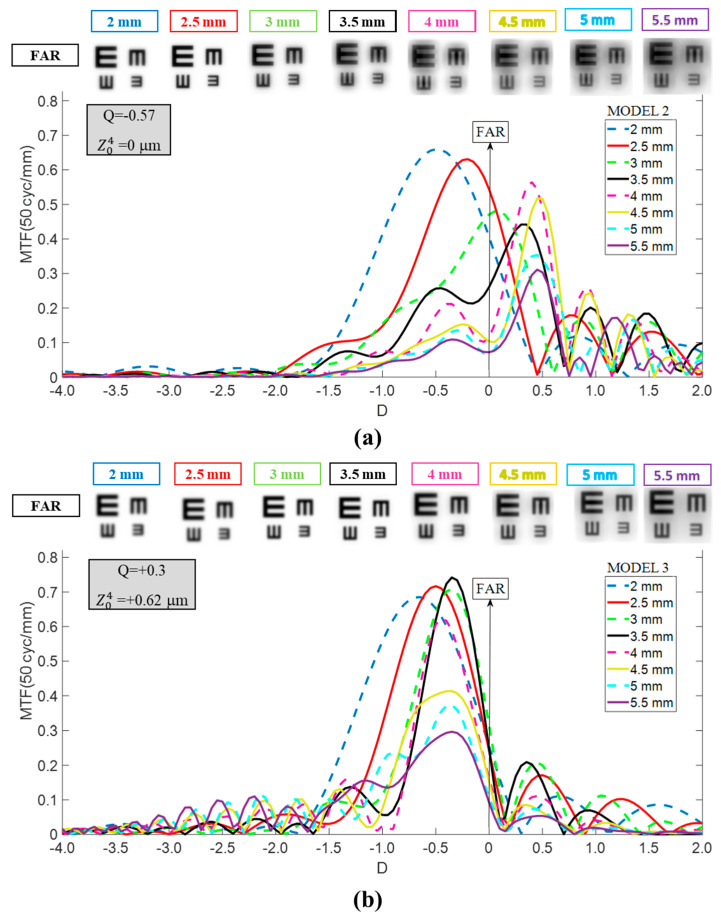
Dependency of the Tecnis Eyhance TO MTF curves on the pupil size for Models 2 (**a**) and 3 (**b**). Simulated optotypes for far vision and all pupil sizes are shown.

Compared to Model 1, the TO-MTFs of the Eyhance in Model 2 shifted toward the hyperopic zone, accompanied by a decrease in the main peak value and a slight increase in the plateau values (Figure 5). These changes were more prominent as pupil size increased. Conversely, for Model 3, a −0.3 D to −0.5 D myopic shift along with a narrowing of the curves was observed (Figure 5, lower panel) compared to Model 1.

## 4. Discussion

Initially, we analyzed IOL behavior using an eye model with a mean population cornea (Model 1). As shown in Figure 2, the Mini Well exhibited marked pupil size dependence, displaying two distinct behaviors. For smaller pupils (2–2.5 mm), the TO MTF presented a single, broad peak shifted myopically. These findings were consistent with those reported by Lee et al. for a 2 mm pupil [17]. Under these conditions, intermediate vision remained adequate, near vision was acceptable, and far vision was impaired. For larger pupils, the Mini Well provided a DOF zone from intermediate to near distances, plus an asymmetrical far peak merging with the intermediate zone. Consequently, optotypes remained recognizable at near and intermediate distances for pupil sizes up to 3.5 mm, but the quality in the DOF zone deteriorated with larger pupils, due to fragmentation into multiple lobes. Conversely, far vision remained acceptable—and even improved—as pupil size increased.

Regarding the Eyhance (Figure 4), with Model 1 and pupils under 3 mm, the TO MTF also displayed a single, broad peak shifted myopically, although less strongly. This behavior and the shift magnitude aligned with the findings reported by Vega et al. [18]. Optimal performance was at sizes 3.0 and 3.5 mm, where the myopic shift was negligible, and a plateau appeared that extended almost to the end of the intermediate zone. For larger pupil sizes, the far vision peak narrowed, resembling a monofocal IOL. This trend for larger pupils has been previously reported in certain studies [18,19], specifically for a pupil diameter of 4.5 mm. Consequently, the Eyhance exhibited adequate far vision irrespective of pupil size, although intermediate vision at 60 cm was acceptable only with 2.5–3 mm pupils, and up to 3.5 mm at 90 cm.

In step two, we examined the IOL’s behavior in Model 2, with a more prolate anterior corneal surface (Q = −0.57 vs. −0.18). This configuration aligns with ISO-1 [1] and represents patients whose asphericity and corneal SA lay at the lower end of the range observed in the normal population [20,21], as well as corneas that have undergone hyperopic LASIK [22]. As shown in Figure 3, the shape of the Mini Well’s TO MTF curves closely resembles those previously reported [23,24], but shifted hyperopically. This discrepancy arises because, in those studies, the axis origin (0 D) was defined at each pupil size’s best far-vision focus using an optical bench with the ISO-1 model eye. By contrast, in our study, we established this reference where the MTF at 50 cycles/mm for a distant object peaked, using Model 1 with a 3.5 mm entrance pupil. This methodological difference enabled us to demonstrate that a patient with average biometric values but a more prolate cornea requires special consideration when selecting IOL power. Most calculation formulas ignore corneal asphericity, so Models 1 and 2 would otherwise yield the same recommended IOL power. However, as Figure 2 and Figure 3 (top panel) illustrate, this choice would incur residual refractive errors of +0.5 D to +0.7 D for pupils exceeding 3 mm. Regarding the TO MTF profile, both models exhibited similar tendences, yet Model 2 presented a slightly softer and broader DOF region. These findings also would apply to post-hyperopic LASIK patients.

For the Eyhance, compared to Model 1, the curves shifted toward the hyperopic region, accompanied by a decrease in the main peak value and a slight increase in both the plateau values and their extent (Figure 5). In a recent study, Kozhaya et al. [25] assessed how controlled SA affects distance-corrected visual acuities for distant (CDVA), intermediate (DCIVA), and near (DCNVA) in different IOLs. They found that reducing SA improves the Eyhance’s DCIVA but diminishes CDVA. These observations align with our findings when transitioning from Model 1 to Model 2 (i.e., a reduction in SA). The worsening of CDVA is related to the decrease in the main peak value, whereas the improvement in DOF is associated with the higher and wider plateau.

Finally, Model 3 was generated by making the cornea more oblate (Q = +0.3), raising SA to +0.62 μm (6 mm of pupil entrance), which is beyond ISO norms but representative of post-myopic LASIK [22] or highly positive SA corneas [20]. To the best of our knowledge, there are currently no published studies available for direct comparison. A more positive corneal SA induced a myopic shift (≈−0.5 D for pupil diameters >3 mm) in the Mini Well’s TO MTF curves relative to Model 1 (Figure 3, lower panel), accompanied by a narrowing that reduced the DOF. Similarly, for the Eyhance (Figure 5, lower panel), a myopic shift with a narrowing of the curves was observed. These outcomes were consistent with previous studies on patients who underwent myopic corneal refractive surgery before multifocal IOL implantation [26,27,28], which concluded that greater corrected myopia (i.e., more positive ΔQ) results in higher residual refractive error post-cataract surgery [28]. Our findings confirm this pattern, as the shift is notably larger when transitioning from Model 2 to Model 3 (ΔQ ≈ +0.87) than from Model 1 to Model 3 (ΔQ ≈ +0.48).

**Figure 6:**
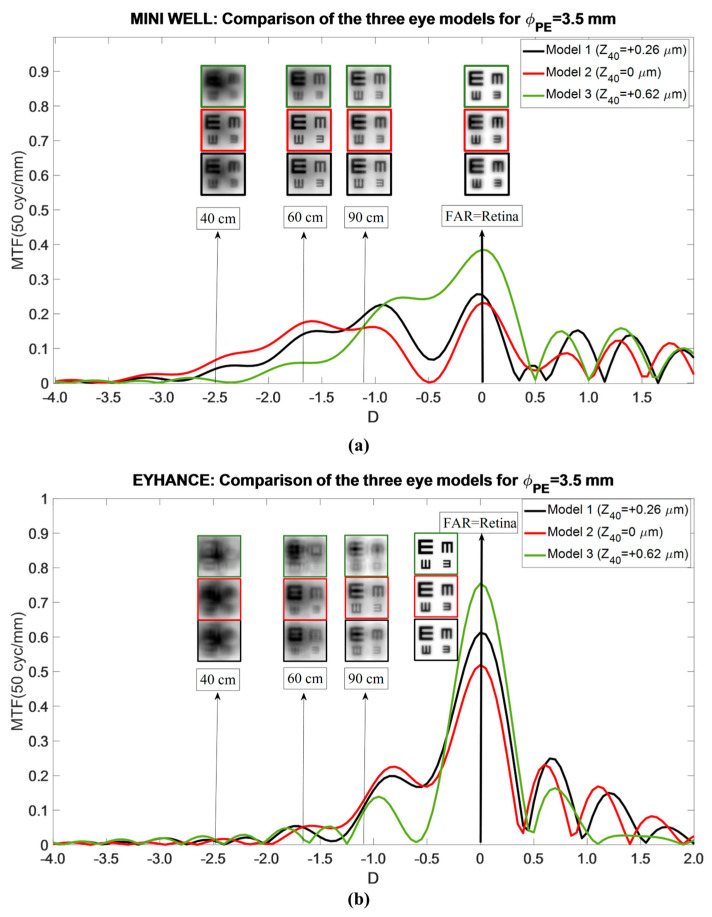
Mini Well (**a**) and Tecnis Eyhance (**b**) TO MTF curves for the 3.5 mm pupil size and the three Models (black color for Model 1, red color for Model 2 and green color for Model 3). An optimized IOL power calculation was assumed to obtain the image of a far object on the retina. Simulated optotypes at far distance, 90 cm, 60 cm, and 40 cm are also shown.

Important questions to be answered would be whether the position of the far focus was compensated by the IOL power adjustment, and whether any shift was avoided. This effect can be easily analyzed by performing both IOLs under optimal conditions, defined by a 3.5 mm entrance pupil and IOL power adjustment to prevent far-focus shift across different asphericity profiles (see Figure 6). In the Mini Well IOL (Figure 6a), the results indicated that an increase in SA (Model 3) significantly degraded optical quality at near and intermediate distances. Notably, the Mini Well demonstrated greater tolerance to shifts toward lower positive SA values (Model 2) or more prolate corneal profiles. Consequently, although the Mini Well demonstrated increased sensitivity to these shifts for far vision, the selection of adequate IOL power would mitigate these effects. In addition, Figure 6 confirms the previously shown result that Model 2 has a slightly softer and broader depth of field (DOF) region. As can be seen, near vision is better in Model 2 than in Model 1, while maintaining the same quality of intermediate vision. The Eyance IOL (Figure 6b) exhibited distinct performance across the three models. Models 1 and 2 showed enhanced monofocal characteristics, with good far VA and acceptable acuity at 90 cm. Simulated optotypes at 60 cm remained discernible, although with reduced contrast. In comparison, Model 3 behaved more like a conventional monofocal IOL, providing good visual quality only at far distances.

Although the main objective of this study was to evaluate the optical performance of two premium refractive IOLs—one extended depth of focus (EDOF) design (Mini Well) and one enhanced monofocal IOL (Tecnis Eyhance)—in relation to pupil size and corneal SA, it is important to note that for complex optics such as these, as with trifocal IOLs, additional factors may affect retinal image quality. One such factor is the decentration of the IOL relative to the visual axis. The literature consistently shows that trifocal IOLs are more susceptible to optical degradation when decentered, while EDOF and monofocal IOLs tend to tolerate decentration better, with less impact on visual quality [9,29]. Nevertheless, there is no standardized method for measuring decentration in clinical studies, and several investigations suggest that the human eye can tolerate mild to moderate decentration reasonably well [30].

## 5. Conclusions

This study demonstrated that both pupil size and corneal SA significantly influenced the real-world performance of the IOLs analyzed. For patients with average corneal SA and pupil sizes between 3.0 and 3.5 mm, our results suggested that the Mini Well provided acceptable visual quality from far to near distances, whereas the Eyhance struggled to maintain visual quality beyond intermediate distances (90 cm).

For patients with lower-than-normal corneal SA (i.e., more prolate corneas, such as those seen post-hyperopic LASIK), both IOLs exhibited a hyperopic shift in far focus, along with a slight improvement in their respective DOFs. Conversely, for patients with higher-than-normal corneal SA (i.e., more oblate corneas, such as those seen post-myopic LASIK), the shift occurred in the myopic direction, accompanied by a clear reduction in their respective DOFs. Furthermore, although the far-focus position could be offset by adjusting the IOL power, the overall curve shape still reflected the interaction between corneal and IOL SA at a given pupil size. The Mini Well was more tolerant of changes to less positive SA values, while Eyhance behaved like a monofocal lens for more positive SA values.

Finally, surgeons should consider each patient’s corneal asphericity and typical pupil diameter when selecting and calculating the power of the premium IOLs studied, particularly in patients with a history of refractive surgery.

## Data Availability

The data supporting the findings of this study are available from the corresponding author upon reasonable request.

## Abbreviations

The following abbreviations are used in this manuscript:

IOL	Intraocular Lens
SA	Spherical Aberration
EDOF	Extended Depth of Focus
MTF	Modulation Transfer Function
TO MTF	Through-Object Modulation Transfer Function
DOF	Depth of Focus
VA	Visual Acuity
CDVA	Distance-Corrected Visual Acuity
DCIV	Distance-Corrected at Intermediate Distance Visual Acuity
DCNVA	Distance-Corrected at Near Distance Visual Acuity
